# Editorial: Advance in vascular anomalies of head and neck region: from bench to bedside

**DOI:** 10.3389/fneur.2024.1529950

**Published:** 2024-12-20

**Authors:** Deming Wang, Yuchen Shen, Shancai Xu, Yu Cai, Lixin Su, Wayne F. Yakes, Xindong Fan

**Affiliations:** ^1^Vascular Anomaly Center, Department of Interventional Therapy, Shanghai Ninth People's Hospital, Shanghai Jiao Tong University School of Medicine, Shanghai, China; ^2^Department of Neurosurgery, The First Affiliated Hospital of Harbin Medical University, Harbin, Heilongjiang, China; ^3^Department of Oral and Maxillofacial Surgery, School and Hospital of Stomatology, Wuhan University, Wuhan, China; ^4^The Yakes Vascular Malformation Center, Englewood, CO, United States

**Keywords:** vascular anomalies, vascular tumor, vascular malformations, hemangioma, signaling/signaling pathways, treatment, translational research

We are pleased to present our Research Topic, which includes a total of 10 articles, discussing the state-of-art on vascular anomalies' research.

Vascular anomalies are abnormalities or disorders of the vascular or lymphatic system, with a relatively higher prevalence in the head and neck region. According to the International Society for the Study of Vascular Anomalies (ISSVA), vascular anomalies are classified as either vascular tumors or malformations (1). Vascular tumors can be benign, locally aggressive, or malignant. Hemangioma is considered the most common type of benign vascular tumor, which is divided into infantile and congenital hemangioma; given the onset time, both are endowed with unique natural history. Other benign vascular tumors include tufted angioma, pyogenic granuloma, spindle-cell hemangioma, and intravenous lipomas, etc. When it comes to locally aggressive tumors, kaposiform hemangioendothelioma is prone to cause thrombocytopenia among affected infants and children, known as the Kasabach-Merritt phenomenon. Malignant tumors include angiosarcoma, epithelioid hemangioendothelioma, etc.

Regarding vascular tumors, Fernández-Alvarez et al. contributed valuable perspectives on the intravenous lipomas of the head and neck through an up-to-date literature review and summary.

Different from vascular tumors, vascular malformations mostly occur congenitally. In the light of the hemodynamics, vascular malformations can be sorted into low-flow (capillary malformation [CM], venous malformation [VM], and lymphatic malformation [LM]) and high-flow (arteriovenous fistula [AVF] and arteriovenous malformation [AVM]). According to ISSVA, vascular malformations are divided into the following types: simple, combined, vascular malformations of major named vessels, and vascular malformations associated with other anomalies.

For low-flow vascular malformations, Azmoun et al., Zhang et al., and Sun et al. have introduced novel sclerosants on VMs; Yuan and Wang contributed their experiences on the electrochemical therapy combined with injection of pingyangmycin treating VMs. Yang et al. discussed the efficacy of surgical resection, sclerotherapy, and the combination of the two in treating LMs.

For high-flow vascular malformations, Han et al. reported the efficacy and safety of embolization among scalp AVFs. Shen et al. demonstrated the promising prognosis of peripheral AVFs after coil-assisted ethanol embolization. Furthermore, Su et al. shed light on the presentation and countermeasures related to the cardiopulmonary collapse induced by ethanol embolization.

Thanks to the significant development of molecular genetics, our understanding of vascular malformations has gradually shifted from a macroscopic level to a microscopic one. Nowadays, gene mutations are the broadly acceptable etiology of vascular malformations and related syndromes, including somatic or germline types. For example, cutaneous or mucosal CM is considered the somatic mutation of *GNAQ*/*GNA11*; (2) common VM is caused by somatic mutation of *TEK*, whereas sporadic AVM results from *MAP2K1* somatic mutation (3, 4). Some genetic diseases, such as hereditary hemorrhagic telangiectasia or capillary malformation-arteriovenous malformation, happen due to the germline mutation of (*ENG, ACVRL1*, and *SMAD4*) and (*RASA1* and *EPHB4*), respectively (5, 6). Here, Solomon and Comi summarized the latest progress of Sturge-Weber syndrome, most commonly associated with a *R183Q* somatic mosaic mutation in the gene *GNAQ*, from a translational perspective.

Given the complex category and comprehensive system of vascular anomalies, scientists, and clinicians are still facing tremendous challenges in basic, clinical, and translational research. Nevertheless, the knowledge base related to vascular anomalies continues to accrue and further studies are needed to allow for the treatment optimization of these challenging conditions.
